# Galileo—an Artificial Intelligence tool for evaluating pre-implantation kidney biopsies

**DOI:** 10.1007/s40620-024-02094-4

**Published:** 2024-10-02

**Authors:** Albino Eccher, Vincenzo L’Imperio, Liron Pantanowitz, Giorgio Cazzaniga, Fabio Del Carro, Stefano Marletta, Giovanni Gambaro, Antonella Barreca, Jan Ulrich Becker, Stefano Gobbo, Vincenzo Della Mea, Federico Alberici, Fabio Pagni, Angelo Paolo Dei Tos

**Affiliations:** 1 Department of Medical and Surgical Sciences for Children and Adults, University of Modena and Reggio Emilia, University Hospital of Modena, Modena, Italy; 2 Department of Medicine and Surgery, Pathology, IRCCS Fondazione San Gerardo dei Tintori, University of Milano-Bicocca, Monza, Italy; 3 Department of Pathology, University of Pittsburgh, Pittsburgh, PA USA; 4 Division of Pathology Humanitas Cancer Center, Catania, Italy; 5 Division of Nephrology, Department of Medicine, University of Verona, Verona, Italy; 6 Pathology Unit, Città della Salute e della Scienza di Torino University Hospital, Turin, Italy; 7 Institute of Pathology, University Hospital of Cologne, Cologne, Germany; 8 Department of Translational Medicine, University of Ferrara, Ferrara, Italy; 9 Department of Mathematics, Computer Science and Physics, University of Udine, Udine, Italy; 10 Division of Nephrology and Dialysis, Department of Medical and Surgical Specialties, Radiological Sciences, and Public Health, University of Brescia and ASST-Spedali Civili of Brescia, Brescia, Italy; 11 Surgical Pathology and Cytopathology Unit, Department of Medicine-DIMED, University of Padua School of Medicine, Padua, Italy

**Keywords:** Digital pathology, Artificial intelligence, Renal biopsies, Transplant, Pre-implantation biopsy

## Abstract

**Background:**

Pre-transplant procurement biopsy interpretation is challenging, also because of the low number of renal pathology experts. Artificial intelligence (AI) can assist by aiding pathologists with kidney donor biopsy assessment. Herein we present the “Galileo” AI tool, designed specifically to assist the on-call pathologist with interpreting pre-implantation kidney biopsies.

**Methods:**

A multicenter cohort of whole slide images acquired from core-needle and wedge biopsies of the kidney was collected. A deep learning algorithm was trained to detect the main findings evaluated in the pre-implantation setting (normal glomeruli, globally sclerosed glomeruli, ischemic glomeruli, arterioles and arteries). The model obtained on the Aiforia Create platform was validated on an external dataset by three independent pathologists to evaluate the performance of the algorithm.

**Results:**

Galileo demonstrated a precision, sensitivity, F1 score and total area error of 81.96%, 94.39%, 87.74%, 2.81% and 74.05%, 71.03%, 72.5%, 2% in the training and validation sets, respectively. Galileo was significantly faster than pathologists, requiring 2 min overall in the validation phase (vs 25, 22 and 31 min by 3 separate human readers, *p* < 0.001). Galileo-assisted detection of renal structures and quantitative information was directly integrated in the final report.

**Conclusions:**

The Galileo AI-assisted tool shows promise in speeding up pre-implantation kidney biopsy interpretation, as well as in reducing inter-observer variability. This tool may represent a starting point for further improvements based on hard endpoints such as graft survival.

**Graphical Abstract:**

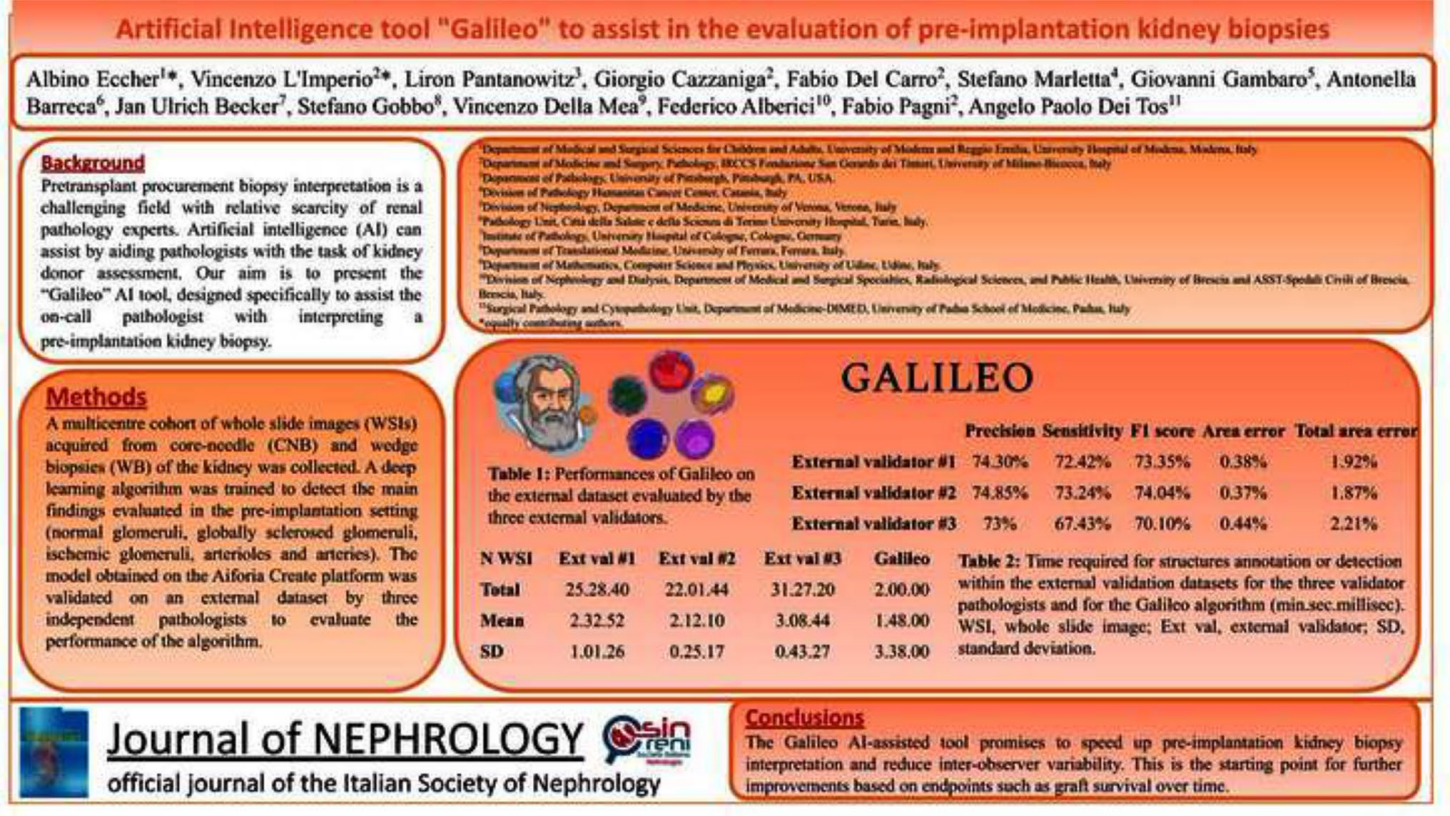

**Supplementary Information:**

The online version contains supplementary material available at 10.1007/s40620-024-02094-4.

## Introduction

In the evolving landscape of kidney transplantation, the quest for precision and consistency in graft evaluation remains a pressing need, especially in the era of extended criteria donors that are boosting the availability of organs with marginal/suboptimal structural characteristics [1]. In this context, the role of pathology in evaluating donor kidneys is crucial in determining the long-term success of transplant procedures [2]. Challenges in transplant pathology include the need for a rapid response during the assessment process and the scarcity of expert pathologists, especially in peripheral centers [3]. Additionally, standardization during sampling phases (needle biopsy vs wedge biopsy), with technical procedures (frozen vs rapid processing) and with reporting protocols, which currently hinge on a multifaceted classification system [4], designed to ensure that only adequate donor kidneys are selected, is lacking  [5]. The Karpinski score provides a systematic and quantifiable assessment of a kidney graft encompassing a range of five parameters focused on glomeruli, tubulo-interstitial compartment and arteries [6]. However, its application in clinical settings is burdened by poor reproducibility and consistency [7].

The construction of a hub-spoke network system with the help of expert renal transplant pathologists can help in improving the diagnostic reliability of the Karpinski score [8], recently facilitated by leveraging telepathology [9, 10] that could further benefit from employing artificial intelligence (AI) tools [11]. Moreover, digitally connecting highly specialized professionals in transplant pathology [12] partly solves the issue of low interobserver reproducibility that affects this score [13]. The application of deep learning tools to speed up pre-implantation biopsy evaluation [14] has shown promising results [15], facilitating the detection, segmentation, and classification of different renal compartments, thereby marking a significant leap forward in precision diagnostics. Herein, we describe the utility and performance of an automated AI-based tool named “Galileo'' that is designed specifically to assist the on-call pathologist with interpreting pre-implantation kidney biopsies.

## Methods

### Case selection

In this multicenter study, consecutive pre-implantation renal histological specimens (*n* = 84) from standard and marginal donors were retrieved from the archives of the Nephropathology Centers of the Department of Translational Medicine, University of Ferrara, Ferrara, Italy (center #1), Fondazione IRCCS San Gerardo dei Tintori, University of Milano-Bicocca, Monza, Italy (center #2) and ASST Spedali Civili, Brescia, Italy (center #3). Of these enrolled cases, 62 were core-needle biopsies processed using a rapid formalin fixation paraffin embedding processing protocol (from center #1), whereas 12 and 10 were wedge biopsies processed using conventional methods (from centers #2 and #3, respectively) [9, 11]. The original Karpinski scores assigned to each case [6] along with clinical information on donors were retrieved at baseline from the hospital electronic health records (age, sex, concurrent diabetes, hypertension, serum creatinine as mg/dl and estimated glomerular filtration rate (eGFR), as calculated with the EPI-CKD formula) [16]. When available, basic clinical and laboratory data on graft outcome (serum creatinine, eGFR and graft survival at 1 year) were recorded. Single Periodic Acid Schiff slides per case were scanned using the NanoZoomer S60 scanner (Hamamatsu, Shizuoka, Japan) at 40 × magnification (0.22079 μm pixel size), fully anonymized and uploaded on the Aiforia Create online platform (v 5.7, Aiforia Technologies, Helsinki, Finland). This study complies with the Declaration of Helsinki and was performed according to ethics committee approval (PNRR-MR1-2022–12375735, 03/16/23).

### Algorithm development and training

**Fig. 1:**
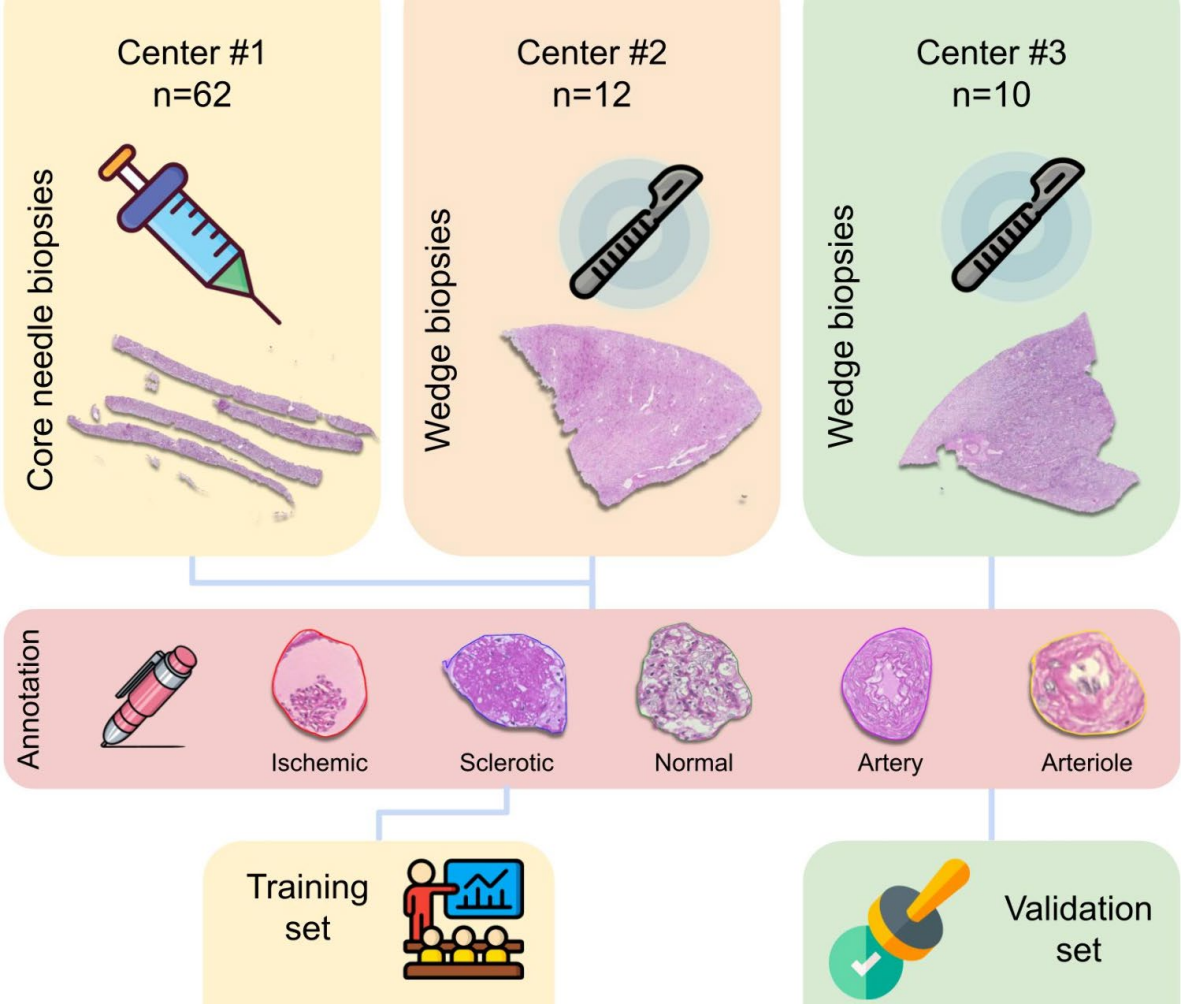
Schematic representation of the pipeline. The case cohort was obtained from different centers in the form of renal CNBs and WBs processed in different ways (rapid vs conventional methods). For the training set, WSIs from the first two centers were comprehensively annotated for 5 object classes and obtained tiles were used for algorithm development. Cases from the third center were included in the external validation set and annotated by three new independent pathologists to evaluate the performance of the model. *CNB* core needle biopsy, *WB* wedge biopsy, *WSIs* whole slide images

For algorithm development, available cases were divided into training and validation datasets, as reported in Fig. 1.

Core-needle biopsy and wedge biopsy cohorts from the first two centers were used as a training set (*n* = 74). Two pathologists (SG and FDC) accurately selected regions of interest from the renal parenchyma containing the five distinct structures employed for this study (normal, ischemic and globally sclerosed glomeruli, as well as arteries and arterioles) from whole slide images collected from the first two centers (Supplementary Fig. 1). Within the selected regions of interest, annotators comprehensively outlined these structures following definitions already provided in the literature [17] using the viewer and annotation tools of the Aiforia platform on a BARCO MDPC-8127 monitor (BARCO, Courtrai, Belgium). All the annotations were subsequently reviewed and refined by two expert nephropathologists (AB and AE). Tiles were subsequently extracted from the annotated images and the AI model was then developed using a field of view of 320 µm, with a training loss of 0.05 and 6032 iterations run. Performance of the final Galileo model was assessed using total area error, precision, sensitivity and* F*1 score (%).

### Algorithm validation

To validate the Galileo model, whole slide images of the wedge biopsies from center #3 (*n* = 10) were used and five regions of interest containing the renal structures under investigation were randomly selected by one of the training annotators (FDC, Supplementary Fig. 2). Within these regions of interest, three additional pathologists (GC, VL and FP, external validators #1, #2 and #3, respectively) independently annotated the different structures providing the ground truth for the validation of the algorithm. Based on subsequent detections by the Galileo model on the validation set, the metrics total area error, precision, sensitivity and* F*1 score (%) were calculated and used to evaluate the performance and generalizability of the deep learning pipeline. Moreover, the time required for the annotation process by external reviewers was recorded with a stopwatch and compared to the time needed for Galileo to detect specific renal structures.

### Statistical analysis

Continuous variables were summarized using mean ± standard deviation (SD), as applicable, while qualitative variables were presented as counts and frequencies. To compare means and qualitative variables, t-tests, chi-square tests, and Mann–Whitney *U* tests were employed, depending on the nature of the data. Comparison of the human and AI evaluation of single parameters of the Karpinski score was performed with Cohen’s kappa (k). Significance was set at *p*-values < 0.05. Collected data underwent statistical analysis using Pandas and Scikit-learn Python libraries.

## Results

### Clinico-histological characteristics of the donors

The study included 84 cases with available pre-transplant biopsy, with a mean donor age of 68 years (± 8.8 years), a prevalence of male donors (67, 80%), 28 (33%) and 34 (40%) of whom had a history of diabetes and/or hypertension, respectively. The average serum creatinine of the cohort was 1.4 (± 0.4) mg/dl corresponding to an eGFR of 54.3 (± 20.1) ml/min/1.73 m^2^. Based on the original histological characterization, cases were divided into two groups, with 39 (47%) and 45 (53%) having a Karpinski score of ≤ 3 and > 4, respectively. A significantly higher prevalence of hypertension (28, 63% vs 6, 14%,  *p* <  0.001), serum creatinine (1.7 ± 0.4 mg/dl vs 1.1 ± 0.2 mg/dl, *p* = 0.001) and lower eGFR at baseline (39.3 ± 10.4 ml/min/1.73 m^2^ vs 71.5 ± 13 ml/min/1.73 m^2^, *p* < 0.001) were present in the latter group (Karpinski > 4), with no significant differences in terms of age, sex and diabetes prevalence (Supplementary Table 1).

### Galileo performance

**Fig. 2:**
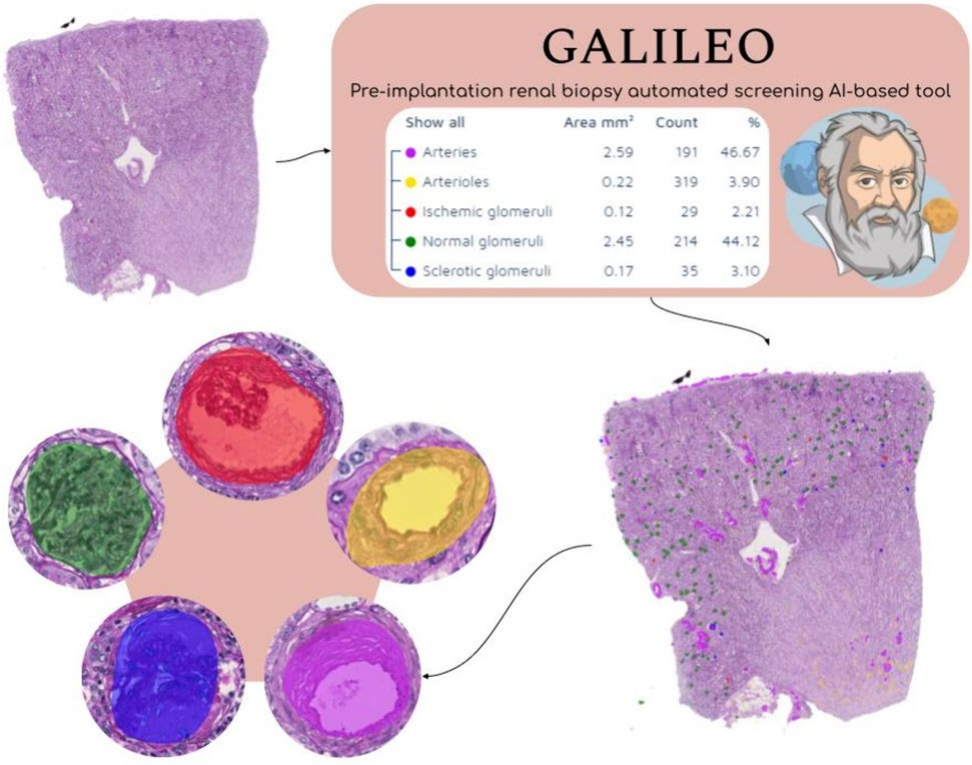
The Galileo algorithm for the detection and classification of renal structures in pre-implantation procurement biopsies. The original WSI is processed with the Galileo AI tool and detections are represented within the tissue in a colorimetric scale (explainable AI) that pathologists can re-evaluate, and numerical as well as percentage measurements are directly extracted and potentially integrable within the final report in the laboratory information system (LIS)

A total of 2880, 79, 506, 587 and 2024 regions of interest representing normal, ischemic glomeruli, globally sclerosed glomeruli, arteries and arterioles were extracted, respectively. The algorithm demonstrated excellent performance during the training phase for 813 training regions, with a precision, sensitivity,* F*1 score and total area error of 81.96%, 94.39%, 87.74% and 2.81%, respectively (Supplementary Fig. 3). The validation phase confirmed the excellent results obtained during training on a separate external dataset (Table 1), with an average precision, sensitivity,* F*1 score and total area error of 74.05%, 71.03%, 72.5% and 2%, respectively. Moreover, the Galileo algorithm was significantly faster as compared to the three external validators, requiring 2 min overall (vs 25, 22 and 31, *p* < 0.001, Table 2), allowing the direct integration of the algorithm results within the final histological report (Fig. 2).

**Table 1:** Performance of Galileo on the external dataset evaluated by three external validators

	Precision	Sensitivity	*F*1 score	Area error	Total area error
External validator #1	74.30%	72.42%	73.35%	0.38%	1.92%
External validator #2	74.85%	73.24%	74.04%	0.37%	1.87%
External validator #3	73%	67.43%	70.10%	0.44%	2.21%

**Table 2:** Time required for structure annotation or detection within the external validation datasets for the three validator pathologists and for the Galileo algorithm (min.sec.millisec)

N WSI	Ext val #1	Ext val #2	Ext val #3	Galileo
1	4.40.13	2.17.19	3.57.14	0.12.00
2	3.02.25	2.20.55	3.13.13	0.10.00
3	1.43.57	1.45.11	2.45.42	0.12.00
4	2.12.45	2.36.07	2.59.28	0.14.00
5	3.59.47	2.00.28	4.26.20	0.10.00
6	1.40.41	1.38.27	1.56.24	0.13.00
7	1.58.53	1.39.29	2.52.58	0.11.00
8	1.58.37	2.28.05	2.32.17	0.12.00
9	2.06.54	2.52.33	3.42.14	0.13.00
10	2.04.28	2.23.10	3.01.30	0.13.00
Total	25.28.40	22.01.44	31.27.20	2.00.00
Mean	2.32.52	2.12.10	3.08.44	1.48.00
SD	1.01.26	0.25.17	0.43.27	3.38.00

*WSI* whole slide image, *Ext val* external validator, *SD* standard deviation

### Graft survival and human/AI reproducibility

One year after transplant, the recipients with functioning kidney transplants had an average serum creatinine of 1.7 mg/dl, with an average eGFR of 68 ml/min/1.73 m^2^. Twenty-two (27%) cases experienced graft loss. Comparing the two groups based on the original Karpinski score (≤ 3 vs > 4), no differences were noted in terms of serum creatinine (1.1 ± 0.7 mg/dl vs 2.2 ± 1.3 mg/dl, *p* = 0.09), but lower eGFR (91.8 ± 38.9 ml/min/1.73 m^2^ vs 47.2 ± 21.2 ml/min/1.73 m^2^, *p* = 0.01) and higher graft loss rate at 1 year (12, 14% vs 32, 38%, *p* < 0.001) were recorded for the latter group. A comparison of the scores given by the pathologist and by the AI system Galileo on the single parameters of the Karpinski score demonstrated good reproducibility for the glomerular, arteriolar and artery scores (*k* = 0.69, 0.65 and 0.67, respectively).

## Discussion

Pre-implantation biopsy plays a central role in kidney graft evaluation and on decisions concerning the possibility to use the kidneys for transplantation. However, the assessment of pre-implantation kidney biopsies is not standardized in terms of the technical procedures adopted and pathologists’ evaluations. Harmonization of this process is needed [4]. Currently, tissue samples may be obtained by core-needle biopsy or wedge biopsy. The most appropriate processing technique (e.g. snap frozen vs rapidly processed) for these specimens is debated [18]. Different policies can have a significant impact on the final report, with possible under-/over-estimation of chronic damage in different renal compartments [19]. This can, in turn, influence the outcome of the graft [20], with the best correlation being described when pre-implantation biopsies are interpreted by experienced renal pathologists [21].

However, the most frequently encountered scenario involves relying on on-call general pathologists, who may have limited knowledge in nephropathology [12]. Moreover, reliance on general pathologists increases inter-observer variability. General pathologists typically assign higher scores for glomerulosclerosis and arterial thickness, which are the most important parameters for evaluating chronic renal damage [8]. To address this challenge, remote teleconsultation by renal experts can be solicited after the biopsy slides are digitized [22]. Once the slides have been scanned, it is also possible to apply computational tools [12]. Indeed, the creation of an AI-based tool that could assist pathologists, by improving accuracy and expediting their review, could be highly beneficial.

The detection of glomerulosclerosis in pre-implantation biopsies is significantly associated with graft survival, with studies demonstrating the predictive role of glomerulosclerosis > 10% [23], with no incremental effects for values above that threshold [24]. This highlights the importance of subtle changes around this cutoff which can be affected by inter-observer variability. Hence, AI-assisted detection of glomeruli, with reliable distinction between normal, ischemic and globally sclerotic, improves diagnostic assessment using whole-slide images [25]. Despite reported challenges with the segmentation and classification task of certain renal structures (e.g. variable shapes/dimensions/internal architecture, interspersed nature within the renal parenchyma, and heterogeneity of pre-analytical variables), previous attempts to apply AI in renal pahology demonstrated high reliability of glomerular detection and classification (e.g. precision in classifying healthy vs sclerosed glomeruli ranging from 0.834–0.935 and 0.806–0.976) [26]. In addition, fibrosis and lumen narrowing of vascular structures (arteries and arterioles) is significantly associated with long term graft survival, especially for mild-moderate (> 25%) arteriosclerosis [24]. Fortunately, AI-assisted segmentation from whole slide images has demonstrated good reliability in discriminating blood vessels vs tubules with an accuracy and precision of 0.93 and 0.88 [27], respectively, confirmed by subsequent studies (accuracy 0.89) which also demonstrated that significantly less time was needed for the algorithm as compared to the pathologists (2 min vs 20 min) [15].

In this study, the Galileo system was trained on a heterogeneous and multi-institutional cohort of renal core-needle and wedge biopsies that included a broad range of pre-analytical variables. The aim was to obtain a robust AI-assisted tool that could be generalized and employed in different settings, to accommodate the heterogeneity of cases encountered in routine clinical practice. Excellent precision and sensitivity were noted for Galileo during the training phase (81.96% and 94.39%), with total area error restricted to only 2.81%. The validation phase on an external dataset annotated by a different panel of pathologists allowed this AI-based tool to achieve good reliability in terms of precision and sensitivity (74.05% and 71.03%), with further reduction of the total area error (2%). Even reaching these promising levels of performance, the AI models can be significantly demanding in terms of computational power, which can potentially limit their wider applications by on-call pathologists due to the potential need for dedicated high performance workstations and to the long computational times [28]. The employment of cloud-based AI suites, like the one used in the present study, can significantly shorten the processing times, i.e., 2 min vs 22–31 for pathologists, which is highly important in the transplantation setting. Another possible limitation of adopting AI could be the reluctance of pathologists in trusting black box solutions [28], which might possibly be mitigated by explainability methods. In this setting, the ability to visually represent renal structures detected by the AI algorithm Galileo in an explainable manner greatly improved end-user acceptance, and facilitated the creation of a final pathology report that integrated qualitative and quantitative findings. Although promising, the current version of the Galileo algorithm includes five histological classes among those required for the interpretation of pre-implantation renal biopsies. The evaluation  of interstitial fibrosis and tubular atrophy (IFTA), not covered by Galileo in its current form, is highly subjective and shows low interobserver reproducibility (Cohen’s kappa of 0.5 among 4 pathologists [29]), which makes it unsuitable for an AI algorithm. Some authors proposed overcoming this subjectivity by quantifying IFTA through image analysis methods, for example using color space transformations and structural feature extraction from the images, that would not need human interaction/training [30]. However, this approach has some limitations including loss of information during the color space transformation, high stain variability (not able to correctly classify all the renal structures), and error in the segmentation of these structures with consequent possible inaccurate quantification of interstitial fibrosis (being based on the identification and subsequent removal of non-fibrotic regions from the tissue). In this setting, application of the adaptive stain separation method seems promising [15], and similar approaches will be implemented prospectively in the Galileo algorithm. Ancillary histological modifications at the glomerular (e.g. mesangial nodular expansion), vascular (e.g. hyalinosis and thrombotic microangiopathy) or tubular (e.g. acute damage/necrosis) level may be of interest to further refine the stratification of the risk in the transplant setting, especially in deceased donors. Further training on larger case series with rare histological instances will be carried out to allow Galileo to recognize ancillary but useful modifications of the renal parenchyma. Moreover, further applications of the Galileo algorithm on additional external datasets will help corroborate its reliability and generalizability in the routine clinical setting, as well as its impact on outcomes (e.g. graft survival).

## Supplementary Information

Below is the link to the electronic supplementary material.

Supplementary file1 (DOCX 12267 KB)

## Data Availability

Authors agree to make data and materials supporting the results or analyses presented in their paper available upon reasonable request.
