# Supplementary material for: Galileo—an Artificial Intelligence tool for evaluating pre-implantation kidney biopsies
Source: J Nephrol. 2024 Oct 2;38(4):1163–9. doi: 10.1007/s40620-024-02094-4 (PMC12187827; doi:10.1007/s40620-024-02094-4)
Supplement: Supplementary file 1 — Supplementary file1 (DOCX 12267 KB) [file 40620_2024_2094_MOESM1_ESM.docx]

**Supplementary Figure 1:** Example of a renal biopsy case annotated for algorithm training. ROIs were selected on the whole slide image (**a**, black circles, x0.5). Pertinent structures of interest present within these ROIs were annotated using different colors (**b**, normal glomerulus in green, globally sclerosed glomeruli in blue, arterioles in yellow and arteries in purple, x10).

**a.
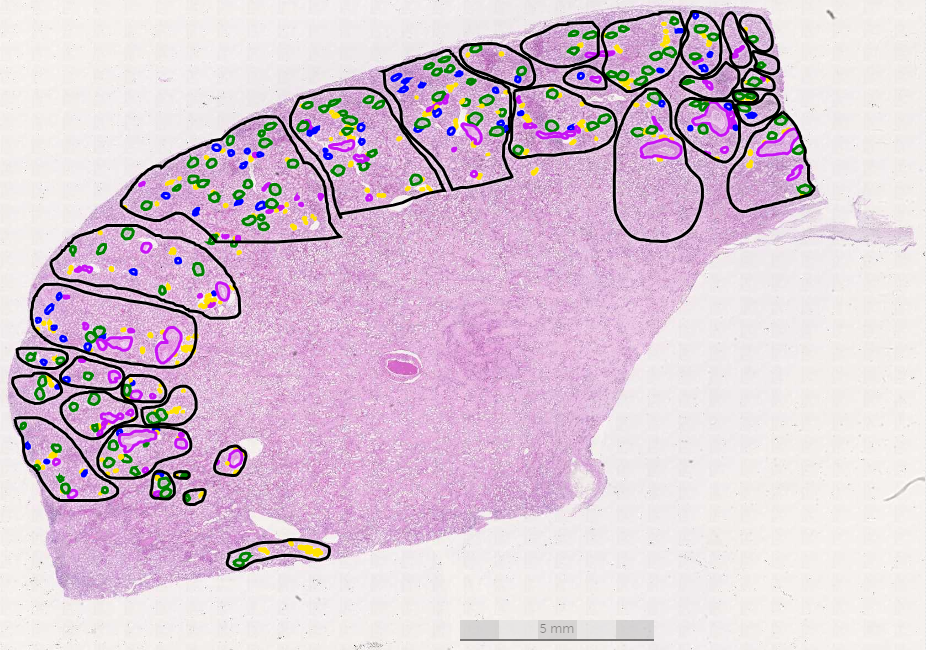
**

**b.
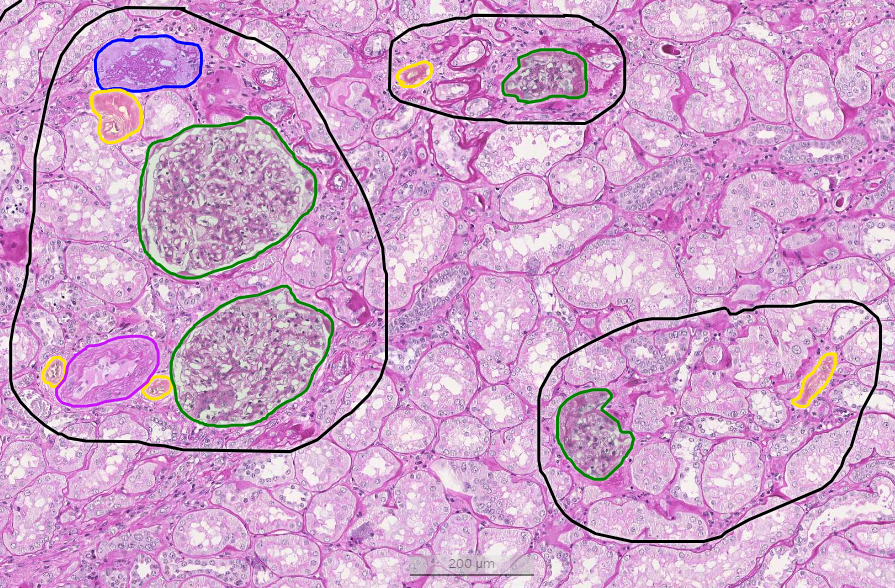
**

**Supplementary Figure 2:** Representation of the validation phase on an external dataset of wedge biopsies from center #3 (n=10). On the WSIs, the original training annotator delineated five ROIs for each slide containing renal structures that can be detected by the algorithm (**a**). Three different pathologists annotated the structures within these ROIs (**b**, normal glomeruli in blue and globally sclerosed glomeruli in green) and performance metrics for the validation phase were extracted from the Galileo algorithm (**c**).

1. **
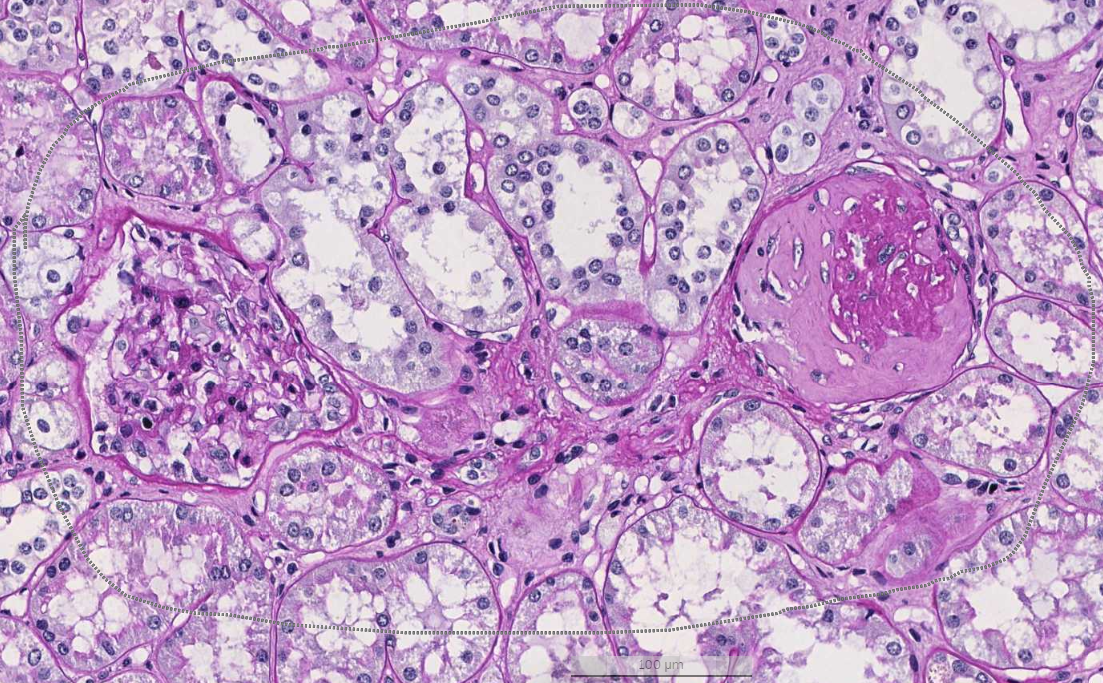
**
2. **
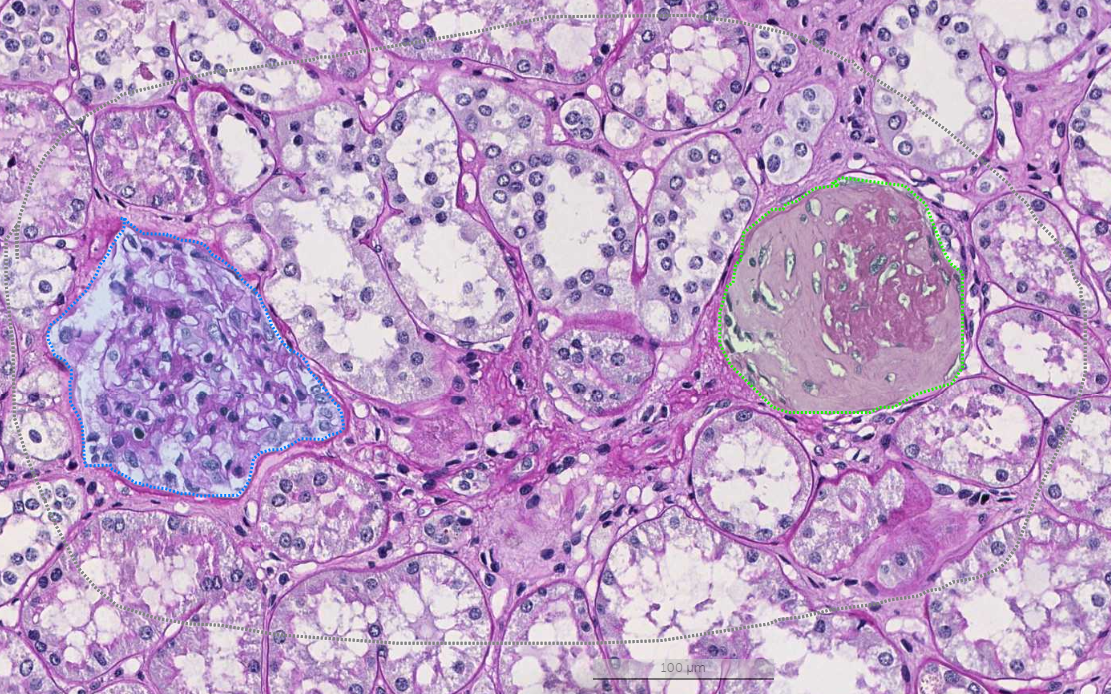
**
3. **
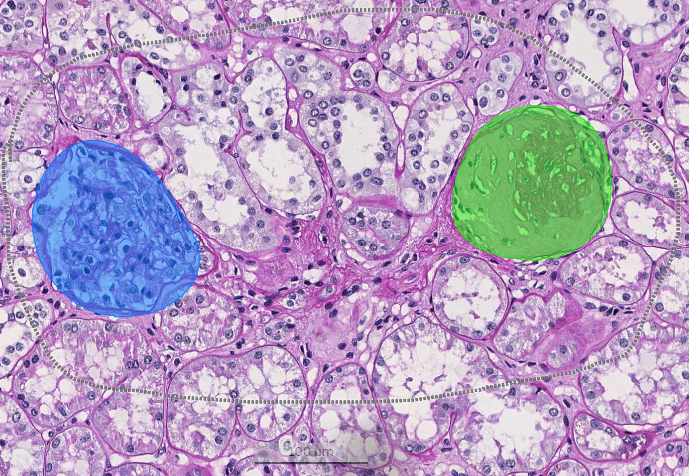
**

**Supplementary Figure 3:** Performances of the Galileo model on the training set. The upper banner shows a WB with the Galileo detections, with the bottom part comparing structure-by-structure the comparability of the AI detection with the corresponding training annotation. WB, wedge biopsy.

**
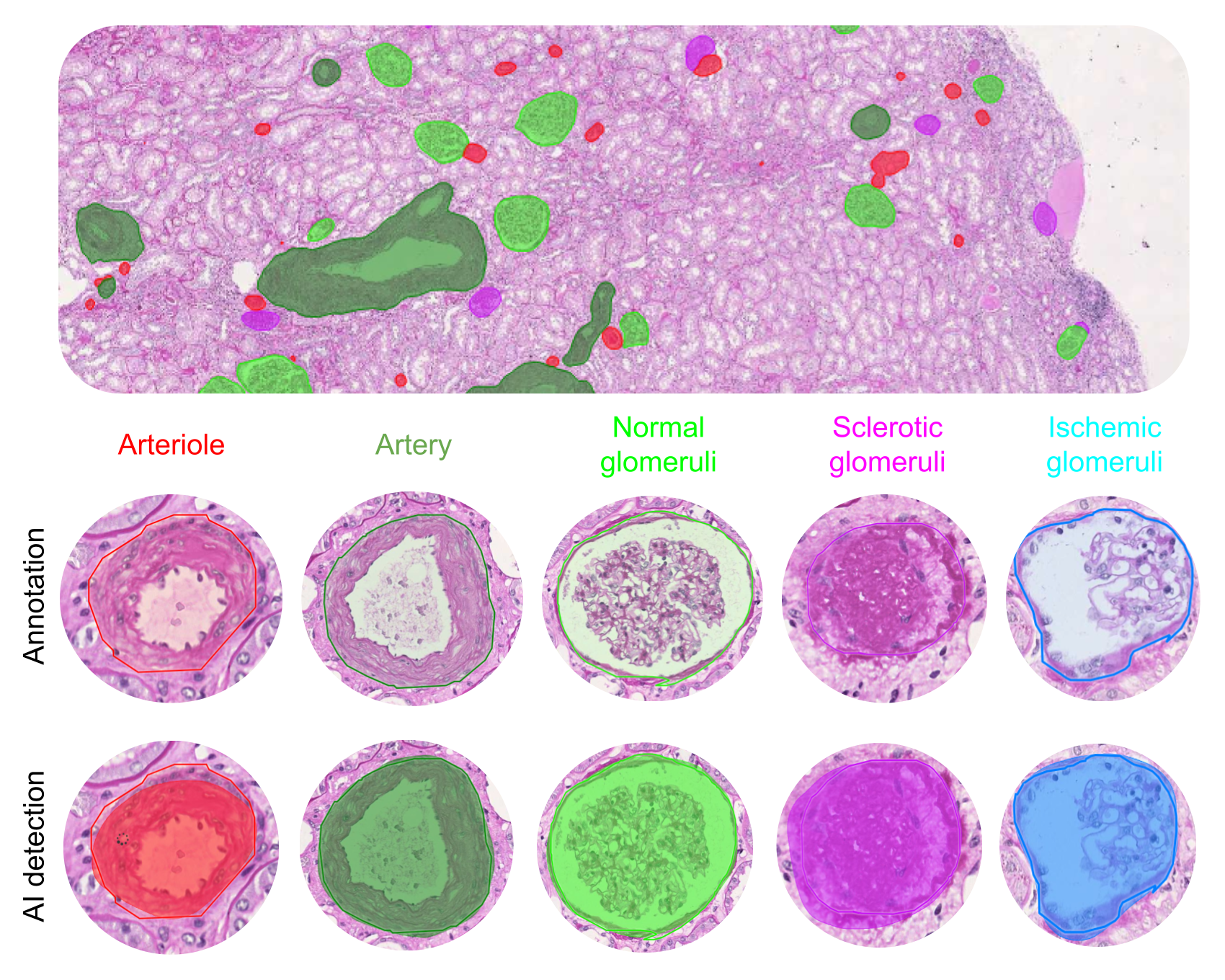
**

**Supplementary Table 1:** Clinico-histological characteristics of the donors. eGFR, estimated glomerular filtration rate.

|  | **Cohort** | | | **Karpinski score ≤ 3** | | | **Karpinski score >4** | | | **p-value** |
| --- | --- | --- | --- | --- | --- | --- | --- | --- | --- | --- |
| **N (%)** | 84 |  |  | 39 | , | 47% | 45 | , | 53% |  |
| **Age (mean ± SD)** | 68 | ± | 8,8 | 65 | ± | 9,0 | 71 | ± | 8,3 | 0,222 |
| **Sex (M, n and %)** | 67 | , | 80% | 34 | , | 86% | 33 | , | 75% | 0,115 |
| **Diabetes (n, %)** | 28 | , | 33% | 11 | , | 29% | 17 | , | 38% | 0,353 |
| **Hypertension (n, %)** | 34 | , | 40% | 6 | , | 14% | 28 | , | 63% | **<0,001** |
| **Serum creatinine**  **(mg/dl, mean ± SD)** | 1,4 | ± | 0,4 | 1,1 | ± | 0,2 | 1,7 | ± | 0,4 | **0,001** |
| **eGFR**  **(ml/min/1.73m2, mean ± SD)** | 54,3 | ± | 20,1 | 71,5 | ± | 13,0 | 39,3 | ± | 10,4 | **<0,001** |
